# The Brazilian Butt Lift: An Analysis of Top-Rated Content on the Social Media Platform TikTok

**DOI:** 10.1093/asjof/ojag098

**Published:** 2026-05-29

**Authors:** Malena Allbright, Mason Horne, Chinenye Ogbonnah, Malcolm Roth, Dzifa Kpodzo

## Abstract

**Background:**

The rising demand for cosmetic gluteal fat grafting is partially attributed to social influence and evolving beauty standards. On the social media platform TikTok alone, content related to what is colloquially known as the Brazilian Butt Lift (BBL) garnered over 15 billion views in the past 3 years, raising concerns about the potential influence of visually striking yet possibly misleading media content on patient decisions and perceptions. This study aimed to analyze the source and content of BBL-related TikTok videos to assess the quality of information presented to potential patients.

**Objectives:**

To characterize top-ranked Brazilian butt lift (BBL) content on TikTok by evaluating (1) creator type and video category, (2) engagement metrics, (3) the quality of educational content using the Patient Education Materials Assessment Tool (PEMAT), and (4) the geographic distribution of patient-reported procedure locations and provider/clinic locations.

**Methods:**

Using the TikTok application, 14 phrases related to the Brazilian butt lift procedure were analyzed. Video analysis included engagement metrics, digital creator type, and video category. The quality of educational content was assessed using the validated PEMAT. A locational analysis of the digital creators was also performed, focusing on the geographic area where patients received their procedures or where physician/cosmetic clinics were located. As an observational content analysis, causal inferences could not be made.

**Results:**

Three hundred fifty videos were included in our study. Patients had the highest percentage of videos (29.1%), followed by “other” (29.0%), and plastic surgeons (20%). Educational videos accounted for the highest percentage of video types (26.6%). Educational videos posted by plastic surgeons had significantly higher understandability and actionability scores than those of non-healthcare creators when using PEMAT (*P* < .001) but had substantially lower views, likes, and saves (*P* < .05). Locational analysis revealed that 77.1% of patient-generated videos with identifiable procedure location referenced procedures reported as having been performed internationally or in Miami (*P* = .021).

**Conclusions:**

The Brazilian butt lift garners high engagement on TikTok. Educational content is common among video subtypes and is high quality when posted by plastic surgeons; however, educational videos receive higher engagement statistics when posted by non-healthcare creators. Content posted by self-identified BBL patients more frequently referenced procedures performed internationally or in Miami; locations that have been associated in prior epidemiologic literature with higher complication and mortality rates in gluteal fat grafting and cosmetic tourism.

**Level of Evidence: 5 (Therapeutic):**

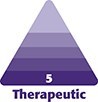

Autologous gluteal fat grafting, better known as the Brazilian butt lift (BBL), has become one of the most sought-after cosmetic procedures over the past decade.^1^ This shift reflects a changing ideal body type, from the slim, athletic look of the 1990s to a curvier aesthetic emphasizing the hips and thighs.^2^ According to the American Society of Plastic Surgeons (ASPS), demand for BBLs has increased from 9993 in 2013 to 28,638 in 2022.^3^

Brazilian butt lift is a commonly performed approach to gluteal augmentation, offering combined liposuction-based body contouring with autologous volume enhancement. Alternative methods, such as silicone, acrylate, and biodegradable filler injections, are discouraged mainly due to serious risks, including infection, necrosis, multiorgan failure, and death.^4^ Despite its popularity, the BBL carries significant risk, particularly pulmonary fat embolism (PFE), with an estimated overall mortality rate of roughly 1 in 3000, the highest of any cosmetic procedure.^5^ Among patients who develop fat-embolism syndrome, in-hospital mortality has been reported at 11.8%, underscoring the potential lethality of the complication.^6^ This is often linked to improper fat injection into or near the gluteal musculature, damaging veins or muscle tissue.^7^

Plastic surgery, being highly visual, finds a natural fit in social media, where patients browse before-and-after photos, follow providers, and gather medical knowledge.^8^ It also serves as a key tool for marketing, branding, and education.^9-11^ Research shows that time spent on social media increases interest in aesthetic procedures.^2^ Platforms like TikTok are especially powerful, with over 1.1 billion users globally (150+ million in the USA) and more than 1 billion videos viewed daily.^12,13^ The hashtag #BBL alone has accumulated more than 15 billion views, reflecting extraordinary public engagement.^12^ Its dominant demographic, users aged 18 to 34 years, matches the average age of BBL patients, 33.6 years.^13,14^

While social media can aid education, it also spreads misinformation, as posts are not peer reviewed or fact-checked.^15,16^ During the COVID-19 pandemic, 76% of users relied on social media for health information, but 63% did not fact-check content.^17^ Cosmetic content on TikTok, often posted by nonphysicians, has been shown to be biased and unreliable.^18,19^ A recent systematic review of plastic surgery–related TikTok content found poor educational quality (mean DISCERN = 1.91/5) and higher accuracy among physician-created videos.^20^

The growing BBL demand has sparked additional concerns: medical tourism (seeking cheaper procedures abroad from potentially unqualified providers), aggressive competitive pricing, surgeon fatigue, and untrained individuals performing procedures.^21-23^ A study by Barr et al^24^ reviewed 7500 websites and identified 3077 unique practitioners; only 2396 (77.9%) were board-certified plastic surgeons, while others included non-surgeons such as internists and emergency physicians performing procedures beyond their formal training. This trend, dubbed the “wandering scalpel phenomenon,” was most prevalent in the Southeastern USA, particularly Miami.

To address safety, the Multi-Society Task Force for Safety in Gluteal Fat Grafting issued 2021 guidelines recommending the use of ultrasound during injections, subcutaneous-only fat placement, and limiting surgeons to three BBLs per day.^25^ Reports, however, showed inconsistent compliance, prompting the Florida Board of Medicine to enact emergency legislation mandating these recommendations in 2022.^21,26^

Despite ongoing risks, the BBL remains in high demand. With social media's influence on decision-making, it is critical to understand the quality of information patients consume. A content analysis of Instagram BBL posts found that 79% were by medical professionals but only 12% mentioned surgical risks, highlighting gaps in patient education.^27^ To date, however, limited peer-reviewed data specifically focused on BBL on TikTok have been reported, emphasizing the need for this investigation.^28,29^

This study aimed to characterize top-ranked Brazilian butt lift (BBL) content on TikTok by evaluating (1) creator type and video category, (2) engagement metrics, (3) the quality of educational content using the Patient Education Materials Assessment Tool (PEMAT), and (4) the geographic distribution of patient-reported procedure locations and provider/clinic locations.

## METHODS

This study was determined exempt by the institutional review board at the senior author's institution. Only publicly available TikTok videos were analyzed, and no identifiable private data were accessed.

Fourteen search phrases related to gluteal augmentation (eg, “BBL surgery,” “BBL recovery,” and “BBL journey”) were used to identify videos (Table 1). For each phrase, the top 25 videos returned under TikTok's “Top” tab were collected, representing the platform's algorithmically ranked popular content.^30^ Duplicate videos that appeared under multiple search terms were removed so that each unique video was included only once. All searches were conducted on the same calendar day to ensure a consistent snapshot of content visibility, and a newly created TikTok account was used to minimize algorithmic personalization bias. All included videos were saved to a private collection within the app for later analysis.

**Table 1.** Search Phrase Characteristics

Search phrase	Total views	Total likes	Total comments	Total saves	Total shares	Posted by PRS N (%)
BBL	45,408,700	2,137,117	39,590^a^	112,081	244,312^a^	4 (16)
BBL before and after	37,891,600	2,608,849	20,031	86,967	47,307	4 (16)
BBL doctors	18,545,219	2,120,312	12,730	56,635	44,383	2 (8)
BBL journey	40,234,800	4,275,485	16,819	110,955	43,130	3 (12)
BBL recovery	16,261,770	1,036,010	4417	27,517	17,727	3 (12)
BBL safety	2,427,183	146,586	4416	9399	8490	16 (64)^a^
BBL surgeon	14,197,680	680,397	11,583	36,558	26,606	5 (20)
BBL surgery	88,399,825^a^	5,211,740^a^	35,939	149,419	171,769	3 (12)
Brazilian Butt Lift	10,200,514	750,910	7655	23,220	27,553	2 (8)
Brazilian Butt Lift Surgery	10,200,514	646,100	5703	19,435	10,698	10 (40)
Butt augmentation	34,998,333	2,715,891	10469	565,816	81,599	1 (4)
Butt implant	13,104,769	852,913	4192	11,106	4669	12 (48)
Butt injection	10,539,556	293,997	1672	23,777	4285	3 (12)
Gluteal augmentation	29,117,600	2,731,751	7856	829,444^a^	85,628	1 (4)

PRS, plastic and reconstructive surgeon. BBL, Brazilian Butt Lift.

^a^Indicates the highest value in the column.

For each video, engagement statistics were recorded, including the number of views, likes, comments, saves, and shares. Engagement rate (ER) was calculated as the ratio of total interactions to total views using the following formula: ER = [(likes + comments + shares + saves)/views] × 100.^30^ Higher ER values indicate greater audience interaction and higher user engagement. Mean and median ER values were calculated across all videos.

Each video creator was categorized as a medical doctor (MD or DO), an allied health professional (AHP, eg, physician assistant or nurse practitioner), a patient, a Medispa (eg, clinic or spa business account), or other (a nonaffiliated creator, influencer, or nonmedical content). Medical doctors and AHPs were grouped as healthcare creators, while all others were classified as nonhealthcare creators. Physicians were further subcategorized as plastic and reconstructive surgeons (PRS) or non-PRS physicians. Plastic and reconstructive surgeons were defined as physicians whose plastic surgery board certification was verified using publicly available physician credentialing resources. Non-PRS physicians included cosmetic surgeons from other specialties or unverified providers. Creators whose specialty could not be verified due to insufficient identifying information were classified as non-PRS physicians, consistent with prior analyses of social media content.^27^

Videos were classified into seven content categories: educational, patient experience, before/after, celebrity-related, promotional, fitness, and other. Educational videos included those discussing procedural information, safety, or postoperative care. Patient-experience videos captured personal recovery, travel, and emotional reflections. Before/after videos showcased aesthetic transformations, while celebrity-related content discussed public figures’ surgeries or physical appearance. Promotional content advertised procedures, clinics, or nonsurgical alternatives, whereas fitness videos demonstrated exercises intended to enhance gluteal size. The “other” category included advocacy, parody, or unrelated content. These categories align with frameworks used in previous social-media analyses of aesthetic-surgery content.^18,19^

For patient-created videos, the geographic location of surgery was recorded when identifiable from captions, hashtags, or user profiles and categorized as Miami, United States (non-Miami), international, or unknown. For physician and Medispa accounts, the location of practice was recorded when available and categorized using the same schema (Miami, United States [nonMiami], international, or unknown). “International” referred to any country outside the United States. When a location could not be determined, it was labeled unknown and included in analyses.

Educational videos were further evaluated using the Patient Education Materials Assessment Tool (PEMAT), a validated instrument for assessing the understandability and actionability of audiovisual patient-education materials, but it does not evaluate factual accuracy or medical correctness.^31^ Two independent reviewers (M.H. and M.A.) independently assessed each educational video, and mean PEMAT scores were calculated for analysis. Reviewer agreement was high; 3 of 93 videos (3.2%) showed discrepancies, which were resolved through discussion and consensus.

All statistical analyses were performed using SPSS version 29 (IBM, Armonk, NY). Descriptive statistics were reported as medians with interquartile ranges (IQR) for non-normally distributed data. The Kruskal–Wallis *H* test was used to compare continuous variables such as PEMAT scores and engagement metrics across multiple creator and content categories. When significant differences were found, pairwise comparisons between groups (eg, PRS vs non-PRS physicians or healthcare vs non-healthcare creators) were conducted using Mann-Whitney U tests with Bonferroni correction to adjust for multiple testing. Categorical data, such as geographic distribution by creator type, were compared using chi-square or Fisher's exact tests when appropriate. All tests were two-tailed, and statistical significance was set at *P* < .05.

## RESULTS

### Overall Video Metrics

A total of 350 unique TikTok videos were analyzed across 14 search phrases, amassing 377,827,417 cumulative views, 26,208,058 likes, 183,072 comments, 2,062,329 saves, and 818,156 shares. On average, each video received 1,079,507 views, 74,800 likes, 523 comments, 5892 saves, and 2338 shares, with a mean engagement rate of 4.78%. Median and IQR values are presented in Table 1 to account for the influence of highly viewed outliers. The phrase “BBL surgery” yielded the highest mean views and likes, whereas “BBL” and “BBL surgery” produced the highest number of comments. The search terms “gluteal augmentation,” “BBL journey,” and “BBL doctors” had the highest engagement rates. Notably, the hashtag **#**BBLsafety contained the largest proportion of PRS videos (16 of 25; 64%) yet had the lowest mean total views, likes, shares, and saves, as well as the second-lowest mean number of comments.

### Digital Creator Analysis

Among all analyzed videos, 102 (29.1%) were posted by patients, 99 (29.0%) by creators classified as “other,” 69 (20.0%) by PRS, 35 (10.0%) by Medispas, 33 (9.0%) by non-PRS physicians, and 12 (3.0%) by allied health professionals (AHPs). Nonhealthcare creators, consisting of patients and “other” users, contributed the majority of top BBL TikTok videos (58.1%), whereas healthcare professionals (PRS, other physicians, AHPs, and Medispas) accounted for 41.9%.

The “other” category comprised 6 subtypes: pop-culture commentators, parody or entertainment accounts, unrelated healthcare creators, fitness influencers, advocacy or awareness accounts, and commercial or promotional pages. Although these users were neither patients nor medical professionals, they collectively achieved the highest median views, comments, saves, shares, and engagement rate. Patient-generated videos had the highest median number of likes and the second-highest levels of views, comments, saves, shares, and engagement. In contrast, Medispas and AHPs consistently had the lowest median engagement. Differences across all six creator categories were statistically significant for every engagement metric (*P* < .001).

PRS creators ranked third overall for views, likes, comments, saves, shares, and engagement rate. Compared with non-PRS physicians, PRS videos had significantly higher median views (*P* = .046), saves (*P* = .046), and engagement rate (*P* = .033) (Table 2). When creators were grouped more broadly, non-healthcare users demonstrated significantly greater visibility and engagement across all metrics compared with healthcare professionals in this dataset (*P* < .001).

**Table 2.** Digital Creator Statistics

Digital creator	Views	Likes	Comments	Saves	Shares	Engagement rate %	Total videos (%)
PRS	102,150	2772	43.5	162	97.5	3.2912	69 (19.7)
Non-PRS MD	73,850	1153.5	22.5	151	64	2.5162	33 (9.4)
AHP	32,500	249.5	5.5	61.5	17	2.1357	12 (3.4)
Patient	273,150	7692.5^a^	106.5	417	197.5	4.1046	102 (29.1)^a^
Other	503,250^a^	16,700	125.5^a^	1321^a^	314.5^a^	4.3598^a^	99 (28.3)
Medispa	30,850	298.5	15.5	75	42.5	1.7809	35 (10.0)
*P* value	<.001	<.001	<.001	<.001	<.001	<.001	N/A
Healthcare	134,450	5122.5	72	303.5	138	3.2035	149
Non-healthcare	319,600^a^	11,000^a^	109^a^	758.5^a^	222.5^a^	4.2464^a^	201^a^
*P* value	<.001*	<.001*	<.001*	<.001*	<.001*	<.001*	N/A

PRS, plastic and reconstructive surgeon; AHP, allied health professional.

All values listed are median unless otherwise stated.

^*^Indicates statistical significance.

^a^Indicates the highest value in the column.

### Locational Analysis

Among the 102 patient-generated videos, procedure location was identifiable in 96 (94.1%); 6 (5.9%) did not specify location. Overall, procedure location was Miami in 37 videos (36.3%), international in 37 (36.3%), and the United States outside Miami in 22 (21.6%); 6 (5.9%) had an unknown location (*P* = .021). Given Miami's prominence as a hub for gluteal augmentation, it was analyzed separately from other U.S. regions. Consequently, 74 of 96 videos with identifiable locations (77.1%) depicted procedures performed either in Miami or internationally.

**Figure 1.**
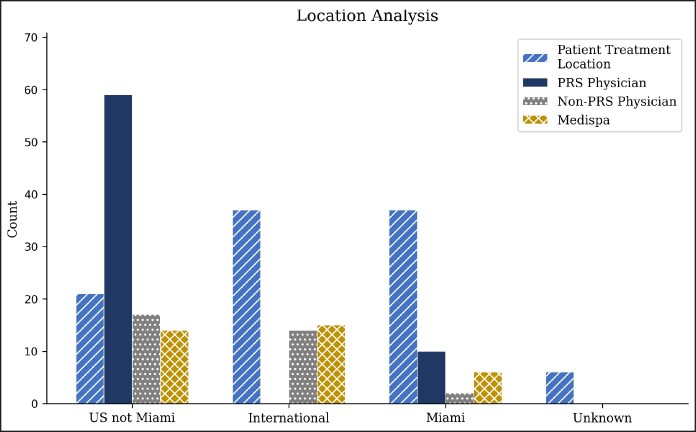
A graph depicting the locations where patients received their Brazilian Butt Lift procedures, as well as the locations of physicians and medispa digital creators. PRS, plastic and reconstructive surgeon.

Among healthcare creators, PRS practice location was USA (non-Miami) in 59 of 69 videos (85.5%) and Miami in 10 (14.5%), with no PRS accounts categorized as international. Non-PRS physicians were primarily international (14/33; 42.4%), followed by USA (non-Miami) (17/33; 51.5%) and Miami (2/33; 6.1%). Medispa accounts were also frequently international (15/35; 42.9%), with the remainder in the USA (non-Miami) (14/35; 40.0%) or Miami (6/35; 17.1%). These distributions are summarized in Figure 1.

### Video Category Analysis

Educational videos comprised the largest category, representing 93 of 350 videos (26.6%). Celebrity-related videos had the highest median number of views and comments but the second-lowest median engagement rate (*P* < .001). Fitness videos demonstrated the highest median likes, saves, shares, and overall engagement (*P* < .001). Although educational content was most common, it ranked second-lowest in median views and likes and third-lowest in engagement rate (Table 3). Fitness and celebrity-related content demonstrated higher engagement metrics than education content in this dataset.

**Table 3.** Video Category Statistics

Type	Views	Likes	Comments	Saves	Shares	Engagement rate %	Total videos n (%)
Before/after	548,050	9671	133	645	464.5	4.0012	46 (13.1)
Celebrity post	987,600^a^	19200	464^a^	1565	503	2.865	13 (3.7)
Educational	81,600	1560	36	158	67	3.2353	93 (26.6)^a^
Fitness	597,250	61,000^a^	201.5	12200^a^	1683^a^	12.1019^a^	26 (7.4)
Humor	314,600	13,800	95	323.5	178.5	3.6635	56 (16.0)
Patient Experience	220,350	5445	86	247	114	3.277	44 (12.6)
Promotional	44,300	427.5	16	85.5	44	1.7345	60 (17.1)
Other	114,550	3298.5	35.5	220.5	60.5	2.2522	12 (3.4)
*P* value	<.001*	<.001*	<.001*	<.001*	<.001*	<.001*	N/A

All values are median unless otherwise stated.

^*^Indicates statistical significance.

^a^Indicates the highest value in the column.

### Educational Content Analysis

The 93 educational videos were further analyzed to assess informational quality using the PEMAT. Most educational videos were created by PRS (n = 44; 47.3%), followed by non-PRS physicians (n = 16; 17.2%), patients (n = 11; 11.8%), and other creators (n = 11; 11.8%). The “other” group achieved the highest median engagement rate (4.7%), followed by patients (4.1%), PRS (3.3%), and non-PRS physicians (2.5) (*P* < .001).

The median PEMAT score for all educational videos was 78.5%. PRS creators achieved the highest median score (90.0%), followed by non-PRS physicians (82.3%), AHPs (66.7%), patients (62.5%), Medispas (60.0%), and other creators (57.8) (*P* < .001). When grouped by creator type, healthcare-affiliated videos demonstrated significantly higher median PEMAT scores than non-healthcare content (84.6% vs 60.0%; *P* < .001). Within the educational subset, engagement metrics did not differ significantly between healthcare and non-healthcare creators, despite significant differences across individual creator categories.

Patient-made educational videos achieved the highest median views, likes, comments, and saves, whereas PRS-authored educational videos, despite their superior quality, had the lowest median saves, second-lowest median views, and third-lowest median likes and comments (Table 4). These results describe differences in engagement metrics across creator types, as well as in PEMAT scores.

**Table 4.** Education Video Sub-analysis

Digital creator	Views	Likes	Comments	Saves	Shares	Engagement rate %	PEMAT % (IQR)	Total videos (%)
PRS	73800	1630.5	29.5	123.5	61	3.2353	90 (81.8-92.3)^a^	45 (48.4)^a^
Non-PRS	92200	1308	37.5	184.5	124^a^	2.8517	84.6 (72.7-88.8)	15 (16.1)
AHP	96100	3302	71	165	23	3.1689	66.7 (50-69.2)	3 (3.2)
Patient	111600^a^	5134^a^	72^a^	307^a^	103	2.8462	62.5 (57.1-71.4)	11 (11.8)
Other	68500	1172	18	150	28	4.6767^a^	57.8 (51.0-60.0)	11 (11.8)
Medispa	82700	834	29	256	43	3.103	60 (58.3-66.7)	8 (8.6)
*P* value	<.001*	<.001*	.412	.019*	.468	.652	<.001*	N/A
Healthcare	81600	1560	35	156	67	3.0481	84.6 (71.4-91.6)^a^	63^a^
Non-Healthcare	92750^a^	3153^a^	62^a^	246.5^a^	71.5^a^	4.1081^a^	60 (54.5-63.6)	30
*P* value	.852	.852	.763	.763	.852	.43	<.001*	N/A

PRS, plastic and reconstructive surgeon; AHP, allied health provider.

All values are median unless otherwise stated.

^*^Indicates statistical significance.

^a^Indicates the highest value in the column.

## DISCUSSION

This analysis of 350 top TikTok videos on Brazilian butt lift demonstrates that BBL content has extraordinary reach and that the creators who dominate engagement are largely nonmedical users. Non-healthcare accounts produced the majority of high-visibility posts, while PRS contributed one-fifth of the content. Educational posts by PRS achieved the highest information quality on the Patient Education Materials Assessment Tool (median 90%); yet, they drew substantially less attention than patient and lay-creator videos, which had lower quality scores. Patient-generated videos frequently referenced procedures reported as having been performed in Miami or internationally. Prior epidemiologic work identifies South Florida as a U.S. outlier for BBL-related mortality, with deaths concentrated in high-volume, budget clinic settings.^21^ International procedure locations in patient narratives align with cosmetic tourism, which prior literature links to serious postoperative complication burden and fatalities among U.S. patients receiving cosmetic surgery abroad.^22,23,26^ Importantly, these risk signals are not inherent to geography and likely reflect systems-level factors described in the literature, including high-throughput clinic models, variability in practitioner training and credentialing, and practice environments that have prompted published safety advisories addressing technique and procedural volume.^21,24,25^ Safety-oriented content, including material tagged with “#BBLsafety,” attracted relatively low engagement, even when produced by specialists, suggesting a misalignment between content quality and audience amplification.

These findings align with and extend prior work on social media and aesthetic surgery. Plastic surgery is inherently visual and lends itself to discovery and branding on social platforms, and time spent on social media correlates with interest in cosmetic procedures.^2,8,9^ TikTok generally delivers higher engagement than Instagram for comparable plastic surgery posts, which gives it outsized influence among potential patients.^11,19,28^ At the same time, multiple studies have shown that health information quality on TikTok is often poor and that physician-generated videos score higher on educational quality than those from non-physicians.^18,20^ Our results mirror these patterns in a high-risk surgical domain and add a key nuance: on TikTok, the accounts that most reliably reach viewers are patients and entertainment-oriented creators, not clinicians.

Comparison with Instagram content further contextualizes these results. On Instagram, a recent analysis of BBL posts reported that most content originated from medical professionals but that only a small fraction disclosed risks, reflecting a marketing-heavy emphasis with limited safety messaging.^27^ In contrast, the present TikTok sample shows a creator mix dominated by nonprofessionals, with safety-focused content underrepresented and under-engaged despite substantial PRS participation. Together, these platform differences suggest that short-form, algorithm-driven discovery may preferentially amplify personal narratives, fitness and celebrity themes, and promotional or sensational elements over fact-forward patient education.

The public health implications are substantial. Patient-generated content is typically positive and can normalize procedures while downplaying risks, which may inflate perceived safety and shape expectations.^9,32^ When women are presented with accurate information about BBL risks, willingness to undergo the procedure falls sharply, underscoring the importance of accessible, evidence-based communication.^33^ Our locational analysis indicates that more than 70% of patient videos in this sample depict surgeries performed in Miami or outside the United States, locales that have been repeatedly linked to higher rates of complications and deaths in gluteal fat grafting and cosmetic tourism more broadly.^21-23,26^ In such environments, high-volume clinic models and variable training standards can contribute to elevated risk, including pulmonary fat embolism, the leading cause of BBL mortality.^5,7^ If highly visible TikTok narratives portray these settings without balanced disclosure, they may shape risk perceptions and influence decision-making.

These observations suggest several actionable implications. Improving the visibility of accurate, comprehensible, and engaging safety information on TikTok should be a shared priority for clinicians, professional societies, and platforms. Thoughtful digital strategies and platform collaborations may help align what is most watchable with what is most trustworthy, thereby reducing preventable harm from a high-risk aesthetic procedure.

First, surgeons and professional societies should expand their presence on TikTok and adapt educational content to the platform's native styles, including concise storytelling, clear visuals, repeated safety cues, and calls to action that encourage verification of credentials and informed consultation.^8,19^ High-quality information alone is insufficient if it fails to capture attention; optimizing for engagement while preserving accuracy should be a central goal. Second, platform-level solutions deserve exploration. Partnerships between TikTok and surgical societies could support verification badges for board-certified accounts, authoritative labels for safety content, or brief interstitial warnings for high-risk procedures, approaches that have been recommended in broader reviews of health information quality on social media.^20^ Third, patient-facing educational materials should be written at appropriate literacy levels and designed for cultural relevance and actionability, given evidence that current BBL information online is often too complex and insufficiently clear.^34^

This study has limitations. TikTok's “Top” results are dynamic and reflect an algorithm that evolves over time, so our dataset represents a snapshot from 1 day and may not capture subsequent fluctuations. Although we used a new account and standardized collection to reduce personalization effects, algorithmic bias cannot be fully excluded. The search strategy included fourteen targeted phrases spanning medical and colloquial terms; yet, it may have missed relevant videos that did not employ these tags. Engagement metrics were captured at a single time point and can change as videos continue to accrue views. Content categorization and quality scoring, while performed by multiple reviewers using a validated instrument that aids in evaluating understandability and actionability, do not directly measure medical accuracy.

Finally, we did not analyze creator follower counts, comment sentiment, or the effects of video length and production style, all of which may influence engagement.

## CONCLUSIONS

In conclusion, TikTok is a powerful venue for BBL information but currently amplifies lay and entertainment content over expert educational material. Plastic surgeons’ videos achieve the highest educational quality yet struggle to reach viewers, while patient and nonmedical posts with lower quality scores dominate engagement. Popular patient videos often feature surgeries performed in international settings or in Miami.
